# Explainable AI: current status and future potential

**DOI:** 10.1007/s00330-023-10121-4

**Published:** 2023-08-17

**Authors:** Bas H. M. van der Velden

**Affiliations:** Wageningen Food Safety Research, Akkermaalsbos 2, 6708 WB Wageningen, The Netherlands

There has been an increasing trend of using artificial intelligence (AI) in high-stakes decision-making that has an impact on human lives, including but not limited to the criminal justice system, autonomous vehicles, food safety, and radiology [1]. The current standard for AI in radiology is deep learning [2]. Deep learning uses neural networks with many interconnected layers that involve nonlinear relationships. Even if we try to understand and describe these layers and connections, it is unfeasible to fully grasp how the neural network makes its decisions. This is why deep learning is often called a “black box.” People are worried that these black boxes might have biases that go unnoticed, which could have serious consequences in high-stakes decision-making [1].

There is a growing demand for methods to improve our understanding of the black box nature of deep learning. These methods are often referred to as explainable artificial intelligence (XAI) [3]. Some notable XAI initiatives include those by the United States Defense Advanced Research Projects Agency (DARPA) and the Association for Computing Machinery’s (ACM) conferences on Fairness, Accountability, and Transparency (ACM FAccT) [4, 5]. For medical imaging, there is a dedicated annual workshop on Interpretability of Machine Intelligence in Medical Image Computing (iMIMIC) at the International Conference on Medical Image Computing and Computer Assisted Intervention (MICCAI) [6].

## Current XAI status

Current XAI techniques in radiology typically either provide a visual explanation, a textual explanation, an example-based explanation, or a combination of these [7]. Visual explanations often provide a “heatmap” or “saliency map,” pinpointing where the algorithm based its decision on. Visual explanations are currently by far the most used XAI technique in radiology [7]. Textual explanations provide textual descriptions, ranging from relatively simple descriptions such as “hyperintense lesion” up to entire medical reports. Example-based explanations provide relevant examples to explain how a neural network made a decision. It is similar to how a radiologist leverages past cases to analyze the case at hand.

Many XAI methods are post hoc, which means that they provide explanations after a neural network has already been trained. This has several advantages [7]. For example, post hoc XAI techniques are often open source and relatively “plug and play,” especially in frameworks such as captum.ai. Furthermore, post hoc XAI is often model agnostic, meaning that it will generate an explanation regardless of the algorithm it is explaining. Therefore, it is possible to provide explanations to neural networks that are currently operational in your clinic or department. There are also some notable disadvantages to post hoc XAI. Post hoc XAI can demonstrate unexpected behavior. For example, not all post hoc XAI techniques demonstrate high validity [8], defined as whether the explanation is correct and corresponds to what the end user expects [7]. Furthermore, there are concerns about robustness [9]. A practical advice to overcome these disadvantages is to examine multiple post hoc XAI techniques and assess the consistency between the explanations.

## Future XAI potential

An important step is to evaluate how well an XAI technique performs. Several evaluation methods exist from computer vision [10], but these do not fully translate to radiology. Therefore, “Clinical XAI Guidelines” have recently been proposed [11] to evaluate XAI techniques in medical images based on five criteria: (1) understandability, (2) clinical relevance, (3) truthfulness, (4) informative plausibility, and (5) computational efficiency. These five criteria were evaluated in radiological tasks for sixteen commonly used visual explanation techniques; none of them met all five criteria [11]. This further reinforces the need for adopting explainable-by-design methods [1], which integrate explainability into AI models from their initial development stages [1].

It is often said that there is an inherent tradeoff between performance and explainability that cannot be avoided. This is not necessarily true: An exciting development is to utilize XAI to improve AI performance [12]. As an example, visual explanations can be used to rank which radiological images should be used next in active learning, leading to a better-performing AI model [13]. This ranking could also be used to select which image to label next, in case of a human-in-the-loop setting with many unlabeled images. Another example uses visual explanations to enforce differentially between visual explanations per class in each sample. This yields better performance, and the visual explanations align more with expert annotations [14].

XAI can be expanded to incorporate biological explanations. As an example, pathway analyses of gene expression data from RNA sequencing revealed that MRI characteristics of breast cancer, such as the contrast enhancement, the smoothness, and the sharpness of the cancer, can be explained by ribosome and peptide chain elongation pathways [15]. This shows the potential of biological processes to be used as explanations.

To go beyond mere correlation and provide explanations that demonstrate cause-and-effect relationships, XAI needs to incorporate causal relationships [16]. By integrating causality in XAI, radiologists can gain a deeper understanding of the underlying mechanisms behind AI-driven decisions. An advantage of incorporating causality is the ability to gain insights into potential biases or to remove such biases [17]. Initial examples of causality in XAI include those using a counterfactual explanation. Let us imagine a chest X-ray showing pleural effusion. How would the same chest X-ray need to appear for the classifier to not predict pleural effusion? This is a counterfactual explanation. Such a counterfactual can provide a personalized and interactive explanation [18].

In summary, explainable artificial intelligence (XAI) is a young, rapidly evolving, and exciting field. It is essential for us as a community to actively contribute to the direction of XAI in the field of radiology. By deciding together on the criteria and aspects that should be prioritized, we can shape the future development of XAI techniques in radiology. This involvement ensures that the emphasis is placed on the specific needs and challenges of the radiology domain, enabling us to create personalized XAI that aligns with the need of clinicians, radiologists, and patients, while complying with regulatory standards [19].

## Declarations

### Guarantor

The scientific guarantor of this publication is Bas van der Velden.

### Statistics and biometry

No complex statistical methods were necessary for this paper.

### Methodology

• Invited commentary
